# Neuroprotective Effects of Green Tea Seed Isolated Saponin Due to the Amelioration of Tauopathy and Alleviation of Neuroinflammation: A Therapeutic Approach to Alzheimer’s Disease

**DOI:** 10.3390/molecules27072079

**Published:** 2022-03-24

**Authors:** Muhammad Imran Khan, Muhammad Zubair Khan, Jin Hyuk Shin, Tai Sun Shin, Young Bok Lee, Min Yong Kim, Jong Deog Kim

**Affiliations:** 1 Department of Biotechnology, Chonnam Notational University, Chonnam, Yeosu 59626, Korea; imranbiotech1@gmail.com (M.I.K.); zobiskhan143@gmail.com (M.Z.K.); geobae@biolsystems.com (J.H.S.); 2 Department Biotechnology and Biomedical Engineering, Kohsar University, Murree 47150, Pakistan; 3 Department of Food Science and Nutrition, Chonnam National University, Gwangju 61186, Korea; shints@jnu.ac.kr; 4 Research Center on Anti-Obesity and Health Care, Chonnam National University, Chonnam, Yeosu 59626, Korea; kmy@jnu.ac.kr; 5 Department of Refrigeration Engineering, Chonnam National University Yeosu, Chonnam 59626, Korea; yb1114@hanmail.net

**Keywords:** taupathy, neurofibrillary tangles, neuroinflammation, neurodegeneration, kinases, SHY-5Y cells

## Abstract

Tauopathy is one of the major causes of neurodegenerative disorders and diseases such as Alzheimer’s disease (AD). Hyperphosphorylation of tau proteins by various kinases leads to the formation of PHF and NFT and eventually results in tauopathy and AD; similarly, neuroinflammation also exaggerates and accelerates neuropathy and neurodegeneration. Natural products with anti-tauopathy and anti-neuroinflammatory effects are highly recommended as safe and feasible ways of preventing and /or treating neurodegenerative diseases, including AD. In the present study, we isolated theasaponin E1 from ethanol extract of green tea seed and evaluated its therapeutic inhibitory effects on tau hyper-phosphorylation and neuroinflammation in neuroblastoma (SHY-5Y) and glioblastoma (HTB2) cells, respectively, to elucidate the mechanism of the inhibitory effects. The expression of tau-generating and phosphorylation-promoting genes under the effects of theasaponin E1 were determined and assessed by RT- PCR, ELISA, and western blotting. It was found that theasaponin E1 reduced hyperphosphorylation of tau and Aβ concentrations significantly, and dose-dependently, by suppressing the expression of GSK3 β, CDK5, CAMII, MAPK, EPOE4(E4), and PICALM, and enhanced the expression of PP1, PP2A, and TREM2. According to the ELISA and western blotting results, the levels of APP, Aβ, and p-tau were reduced by treatment with theasaponin E1. Moreover, theasaponin E1 reduced inflammation by suppressing the Nf-kB pathway and dose-dependently reducing the levels of inflammatory cytokines such as IL-1beta, IL-6, and TNF-alpha etc.

## 1. Introduction

Alzheimer’s disease (AD) is the most frequent type of dementia, characterized by lose of memory, cognition and ability of communication due to abnormal neurodegeneration [1]. About 24 million people are affected worldwide by AD, with 5 million new cases annually. Age is one of the risk factors of AD and dementia, as incidences and prevalence of AD are relatively higher between the ages of 60–90 [2,3,4]. Molecular study of AD revealed the major causes are the deposition of amyloid-β (Aβ) plaques, tau neurofibrillary tangles (NFTs), and neuroinflammation, leading to lesions in the brain, synaptic loss and eventually neurodegeneration [5,6,7]. Hence, inhibition of Aβ, Tau, and neuroinflammation are the therapeutic targets for AD prevention or treatment. Tau is abundantly expressed in neurons of the central nervous system and stabilizes microtubules, which are tracks for motor proteins. Tau also modulates axonal transport of the microtubule [8,9,10,11]. During neuronal development, Tau is upregulated to promote the generation of cell processes [12]. Hyperphosphorylated Tau detaches from the microtubules and forms fibrils in an insoluble form, i.e., paired helical filaments (PHFs) that aggregate and form NFTs [13,14]. Phosphorylation of Tau occurs via a variety of serine/threonine protein kinases, such as glycogen synthase kinase-3β (GSK-3β), cyclin-dependent kinase 5 (Cdk5), extracellular signal-regulated kinase 2 (ERK2), calcium/calmodulin-dependent protein kinase II (CaMKII), and microtubule affinity-regulating kinase (MARK), etc. If Tau is pre-phosphorylated, GSK-3 accelerates the rate of tau phosphorylation by several fold through priming kinases, such as non-proline-directed kinases [15,16,17]. GSK-3β phosphorylates Tau at Ser400; this process is followed by the sequential phosphorylation of Ser396. GSK-3β can also directly phosphorylate Tau at Ser202; however, Thr231 phosphorylation is required for the pre-phosphorylation of Ser235. Hence, Tau is initially phosphorylated by priming kinases, such as non-proline-directed kinases (non-PDK). When GSK-3β activation is enhanced by Aβ, GSK-3β accelerates Tau-Ser396 phosphorylation, which is responsible for PHTs and NHFs, thereby causing AD [18] Aβ and Tau serve as an initiator and an executor of AD, respectively [19]. Current AD therapeutic approaches focus on targeting Tau pathologies. A variety of Tau-targeting drugs have been developed. Tau-targeted drugs that are currently under development as AD therapies include (i) Hsp90 inhibitors, (ii) inhibitors of Aβ-induced Tau phosphorylation, (iii) Tau aggregation inhibitors, (iv) *O*-GlcNAcase inhibitors, and (v) GSK-3β inhibitors.

Studies suggest that the accumulation of Aβ in the brain due to the imbalance of production and clearance is the major cause and driving force of AD. The pathogenic events in AD are multifactorial; identification of the genes involved in pathogenesis of AD have been determined via use of several animal models. Several molecular pathways and genes are involved in the progression and causation of AD. Mutation or impairment of the various kinases, APP, PSEN1, PSEN2, and APOEε4 lead to AD [20].

Microglia cells clustered around amyloid plaques in the brain play a crucial role in clearance of Aβ [21,22]. Microglia cells express TREM2 within the CNS, which functions in binding and clearing Aβ and plays important roles in phagocytosis of amyloid plaques, apoptotic neurons, and neuronal damages [23,24,25,26,27,28,29,30]. Moreover, TREM2 has been demonstrated to be essential in early development, survival, and regulation of microglia metabolism, synaptic pruning, proliferation, and cytokine release [31,32,33,34,35]. Soluble TREM2(sTREM2), produced by protyulytic cleavage of TREM2 by ADAM10 and ADAM17, acts as an immunomodulatory biomarker for neurodegeneration, and its concentrations correlate with the phosphorylated tau level of CSF [26,36,37,38,39,40].

Inflammation also contributes to exacerbation and progression of AD. Several proinflamatory cytokines, e.g., IL-1 and TNF alpha, were reported to be elevated in AD brain. Accordingly, elevations in IL-1 levels are closely associated with AD pathogenesis and neuroinflammation in the AD brain. Inflammation involved in the pathology and cytokine production by microglia cells contributes to amyloid plaque formation [41]. Reactive microglia surrounding amyloid plaques with increased expression of IL-1 is the initial indication of the IL-1 association with AD pathogenesis [42,43]. Various studies have revealed that neuroinflammation plays a fundamental role in the progression of the neuropathological changes associated with AD. Several studies highlighted the elevated level of TNF-a, IL-1b, and IL-6, and TGF-b in AD patients [44]. The NF-kB pathway is the primary regulator of inflammatory responses, responds to proinflammatory stimuli, such as TNF-a or IL-1, and plays a central role in the development of neuroinflammation [45,46]. The current AD treatments cannot alleviate the progression of AD but only slow the worsening of dementia symptoms. Medications to treat the disease, delay its onset, and prevent its development are essentially required.

## 2. Results

**Figure 1:**
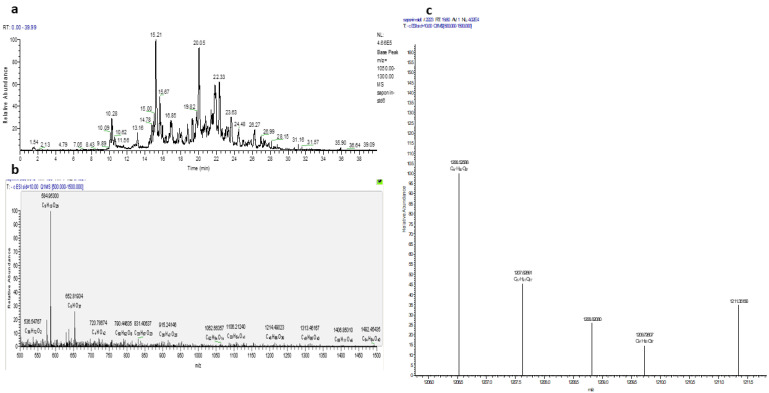
LC-MS-TOF analysis of the green tea seed extracted saponin. (**a**). LC-MS chromatogram of various saponins extracted from green tea seeds via HPLC (**b**) and the purified fraction containing the pure, isolated theasaponin E1 (**c**) The base peak intensity mass spectrum of green tea seed extracted saponins.

Theasaponin E1 was isolated from the saponin-rich fraction of purified green tea seed extract using resin column chromatography followed by preparative HPLC. Subsequently, theasaponin E1 was analyzed with LC/TOF-MS and NMR for identification, structure elucidation, and quantification of the compounds present (Figure 1).

### 2.1. MTT Assay

**Figure 2:**
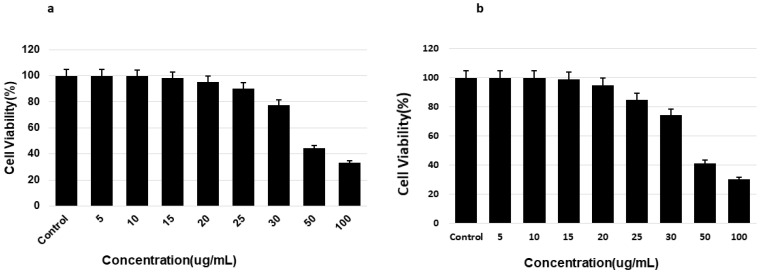
Determination of non-toxic concentration level of theasaponin E1 to SHY-5Y neuroblastoma and glial cells (**a**,**b**), respectively via MTT assay. Cells were cultured in their respective media in a 96-well microtiter plate. After attachment and treatment with saponin, cell viability was determined for each dose by reading the absorbance of the wells after addition of the MTT reagent and calculation of the results. Cells without treatment were employed as the control. Data are shown as mean ± standard error of means (SEM) (*n* = 3).

The toxicity of saponin to SH-5YSY neuroblastoma and HTB-14 glioma cells was determined using an MTT assay. Furthermore, the safe levels of saponin were determined based on the percentage of viable cells. Cells that were not treated with saponins were used as the control and displayed 100% viability. Treatment with 20 μg/mL pure saponin was nontoxic, and greater than 90% cell viability was observed (Figure 2). Increasing the concentration beyond this level led to decreased cell viability and increased toxic effects. The toxicity of saponins to mouse blastoma cells was determined using an MTT assay, and the safe levels of saponins were determined based on the percentage of viable cells. Cells that were not treated with saponins were used as the negative control and displayed 100% viability (Figure 2).

### 2.2. Effects of Saponins on the Activities of Various Kinases and Phosphatases

**Figure 3:**
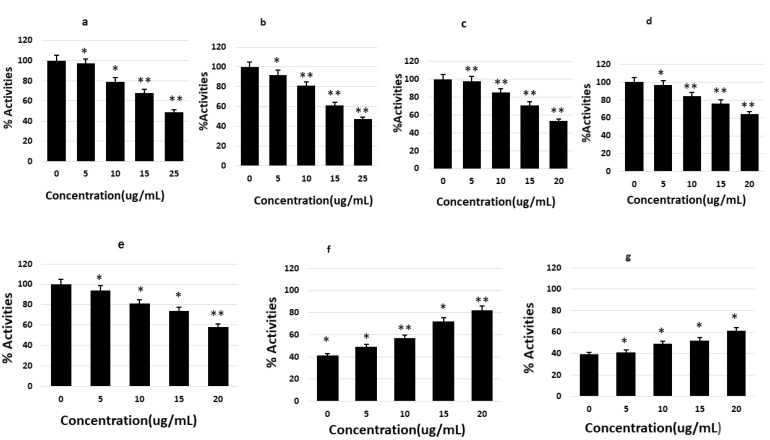
In vitro activities of various kinases and phosphatases involved in tau phosphorylation and dephosphorylation under the effects of theasaponin E1, quantified by ELISA. Percent activity for each enzyme was measured. (**a**) Changes in GSK3beta activity before and after treatment with saponins. (**b**) Changes of CDK5 activity by theasaponin E1. (**c**) Effect of theasaponin E1 on CAMII activity. (**d**) Effect of theasaponin E1 on JNK activity. (**e**) Effects of theasaponin E1 on ERK activities. (**f**) Effects of theasaponin E1 on PP1 activities. (**g**) Effects of theasaponin E1 on PP2B activities. Data are the values of mean ± SEM and were analyzed using one-way ANOVA (*n* = 3) (* *p* < 0.05; ** *p* < 0.01).

The levels of GSK-3β, CDK5, JNK, MAPK, ERK1/MARK, CaMKIIα, PP1, PP2A, and PP2B were determined to derive the inhibitory or activating effects of saponins on Tau phosphorylation. SHY-5Y cells were treated with various nontoxic concentrations of the isolated saponins and the cell lysate was prepared for ELISA. Saponins were found to significantly reduce p-tau by decreasing the activities of the enzymes *GSK-3β*, *CDK5*, *JNK*, *MAPK*, *ERK1/MARK*, and *CaMKIIα* in a dose-dependent manner, with different significant levels. PP1 and PP2A ELISA revealed dose-dependent enhancement of the levels of these proteins compared to their levels in the control (Figure 3).

### 2.3. Gene Expression of Various Kinases and Phosphatases

RT-PCR was used to measure the expression of genes involved in Aβ and p-tau production and their processing to plaque formation NFTs. RNA from cells in the treated and control groups was extracted and reverse transcribed to cDNA. The resulting cDNA was then amplified by RT-qPCR using gene-specific primers (Table 1).

**Figure 4:**
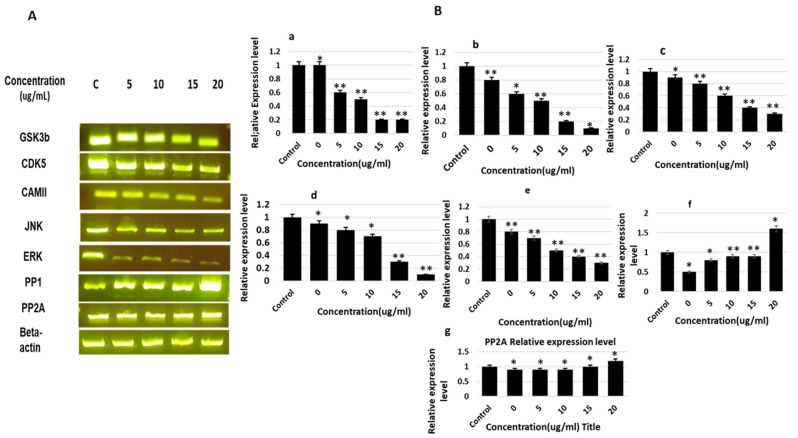
mRNA expression levels of various kinases and phosphatases involved in tau phosphorylation and dephosphorylation, determined by RT-PCR. (**A**). PCR products of mRNA expression of various genes of the kinases and phosphatases presented in gel band form after gel electrophoresis. (**B**). Relative mRNA expression levels of genes of various kinases and phosphatases after treatment with theasaponin E1. (**a**) Relative mRNA expression levels of GSK3 beta. (**b**) Relative mRNA expression levels of CDK5. (**c**) Relative mRNA expression levels of JNK. (**d**) Relative mRNA expression levels of ERK. (**e**) Relative mRNA expression levels of CAMII. (**f**) Relative mRNA expression levels of PP1. (**g**) Relative mRNA expression levels of PP2A. mRNA expression levels of genes of various kinases and phosphatases that were visualized as PCR products on agarose gel. Data are the mean values ± SEM and were analyzed using one-way ANOVA (*n* = 3). Data are statistically significant at *p* < 0.05 (* *p* < 0.05; ** *p* < 0.01 to the control group).

The results revealed significant effects of saponin treatment on the inhibition of several kinases and activation of phosphatases. The expression of the major kinases involved tau phosphorylation (i.e., *CDK5* and *GSK 3beta*), which results in the suppression of tau in a dose-dependent manner. Saponin treatment also affected the expression of ERK and CAMII; however, the levels were found to be comparatively lower than those of CDk5 and GSK3. Moreover, the effect of saponins on the expression of *JNK* was the lowest. Saponin treatment also enhanced the activation and expression of PP1; however, the enhanced expression level was relatively low for PP2A and PP2-B. The relative expression levels of GSK-3β, CDK5, JNK, MAPK, ERK1/MARK, CaMKIIα, PP1, and PP2A were calculated and compared with those of the β-actin control (Figure 4).

### 2.4. Effects of Theasaponin E1 on the Expression of Genes Involved in AD Pathology

RT-PCR was used to measure the expression of genes involved in the Aβ pathway. RNA from treated and control cells was extracted and reverse transcribed into cDNA. The resulting cDNA was amplified by RT-qPCR using gene-specific primers (Table 1).

**Figure 5:**
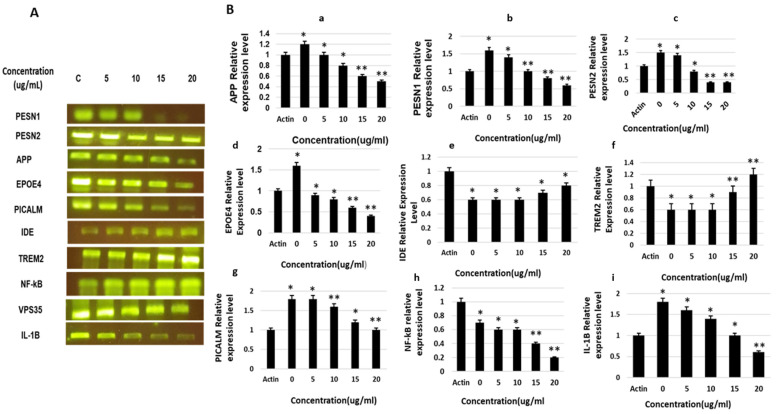
The gene expression of APP, PESN1, PESN2, EPOE4, IDE, VPS35, PCALM, NF-kB, and IL-1B, as under the influence of theasaponin E1, determined by RT-qPCR. (**A**) Expression of APP, PESN1, PESN2, EPOE4, IDE, VPS35, PCALM, NF-kB, and IL-1B, as determined by reverse transcription quantitative PCR, visualized by PCR products bands on membrane after gel electrophoresis. (**B**) Relative mRNA expression levels of various genes. (**a**) Relative protein expression levels of APP. (**b**) Relative mRNA expression level of PESN1. (**c**) Relative mRNA expression level of PESN2. (**d**) Relative mRNA expression level of EPOE4. (**e**) Relative mRNA expression level of IDE. (**f**) Relative mRNA expression levels of VPS35. (**g**) Relative mRNA expression levels of PCALM. (**h**) Relative mRNA expression levels of NF-kB. (**i**) Relative mRNA expression levels of IL-1B. Data are shown as mean ± SEM analyzed with one-way ANOVA (*n* = 3). Data considered statistically significant at *p* < 0.05 (* *p* < 0.05; ** *p* < 0.01).

The results revealed significant effects of saponin treatment on the inhibition of several AD-promoting genes and activation of AD-alleviating genes. The expression levels of the major genes involved in tau and Aβ generation and AD pathogenesis (i.e., *APP*, *PESN1*, *PESN1*, *EPOE4*, and *PICALM*) were significantly suppressed in a dose-dependent manner. Saponin treatment also enhanced the activation and expression of TREM2 and IDE in a dose-dependent manner and affected the expression of inflammatory genes and cytokines involved in AD worsening (i.e., NF-kB and IL-1B). The relative expression levels of all genes were normalized to those of the control (β-actin) and calculated (Figure 5).

### 2.5. Effects of Saponins on Proinflammatory Cytokines

**Figure 6:**
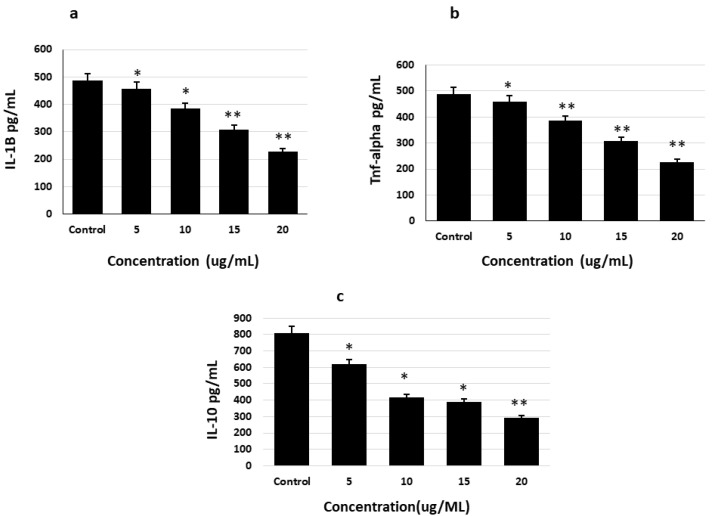
Inhibitory effects of theasaponin E1 on the inflammation-promoting cytokines IL-1B, IL-6, and TNF alpha in glial cells determined by specific ELISA kits. (**a**). Inhibitory effects of theasaponin E1 on IL-1B. (**b**). Inhibitory effects of theasaponin E1 on TNF alpha. (**c**). Inhibitory effects of theasaponin E1 on IL-6. Data shown here are the mean values ± SEM. Data were analyzed using one-way ANOVA (*n* = 3) and were statistically considered significant at *p* < 0.05 (* *p* < 0.05; ** *p* < 0.01 to the control group).

The effects of saponins on the inhibition or suppression of AD pathogenesis were determined by quantifying the levels of inflammatory cytokines (IL-1B, IL-6, and TNF alpha) in glial cells using specific ELISA kits. Saponin was found to dose-dependently decrease the levels of the inflammatory cytokines, IL-1B and TNF alpha, compared to their levels in the untreated control (Figure 6).

### 2.6. Determination of Aβ and P-Tau Levels Using ELISA

**Figure 7:**
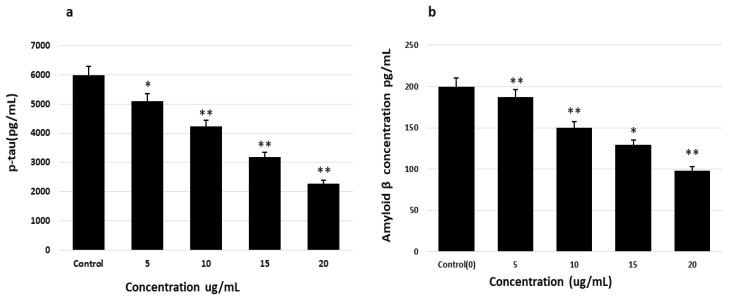
Reduction in the levels of Aβ peptides and p-tau after treatment with theasaponin (due to enhancement or reduction in the activities of various kinases and phosphatases), determined by ELISA. (**a**) Aβ quantification with ELISA after the treatment of cells with different concentrations of theasaponin E1. (**b**) p-tau quantification with ELISA after the treatment of cells with different concentrations of theasaponin E1. Data were analyzed using one-way ANOVA (*n* = 3) and expressed as mean ± SEM. Data are statistically significant at *p* < 0.05 (* *p* < 0.05; ** *p* < 0.01 to the control group).

After determining the effects of saponins on the genes involved in AD alleviation and AD pathogenesis, the Aβ and p-tau levels in cells after treatment with saponins were measured using specific ELISA kits. The levels of p-tau and Aβ were significantly and dose-dependently reduced in treated cells compared to those in the nontreated cells (Figure 7). These findings demonstrate the effects of saponins on the inhibition of p-tau- and Aβ-accumulating and promoting genes and the enhanced expression levels of the AD elevating genes.

### 2.7. Quantification of P-Tau in SH-SY5Y Cells Using Western Blotting

**Figure 8:**
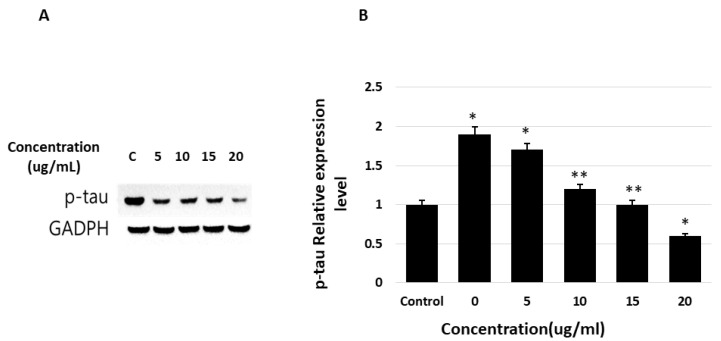
Effects of theasaponin E1 on tau phosphorylation levels determined by WB. Protein expression as fold change relative to that of the control (actin) after normalization. (**A**) Western blotting results after treatment of cells and expression of tau, p-tau, and GAPDH on PAGE. (**B**) Phosphorylated-tau relative protein expression levels determined by western blotting. Data are shown as mean (*n* = 3) ± SEM (* *p* < 0.01 and ** *p* < 0.001 compared to the control).

To determine whether the phosphorylation of tau decreases after the suppression and activation of AD-related genes and inflammatory pathways and cytokines, we verified the concentration of phosphorylated tau in neuroblastoma cells after treatment with saponins, using western blotting. The phosphorylation of tau, a key and crucial element of NFT and AD pathogenesis, was dose-dependently decreased by saponin treatment (Figure 8).

## 3. Discussion

AD is a neurodegenerative disease, the principal cause of which is the abnormal deposition of Aβ and hyperphosphorylated tau, leading to the formation of senile plaques and NFTs in the brain [6].

Hyperphosphorylation of tau may be caused at different locations by various kinases. Under normal conditions, equilibrium exists between tau kinases and tau phosphatases activity. This phenomenon is important for lowering the affinity of tau for microtubules and increasing the resistance of tau to calcium-activated neutral proteases and its degradation by the ubiquitin-proteosome pathway [47]. Fibrillization and aggregation of tau due to tau hyperphosphorylation eventually produce NFT [48,49,50,51,52]. The major tau kinases include GSK-3β, CDK5, PKA, MAPK, CaMK II, and MARK [52,53,54,55,56,57]. Among the phosphatases, protein phosphatase 2 (PP2A) has been most commonly implicated in the dephosphorylation of abnormal tau [58]. Notably, changes in the expression and/or activation of tau kinases and tau phasphatases have been well documented in AD and related disorders [59,60,61,62]. Studies in transgenic mouse models of AD suggest that multiple, overlapping processes might contribute to abnormal hyperphosphorylation of tau, including Aβ, impaired brain glucose metabolism, and inflammation [63,64,65].

Green tea plant *(Camellia sinensis)* is enriched with several bioactive compounds, including saponins, and possess crucial medicinal effects. In the current study, we extracted and isolated pure theasaponin E1 from green tea seeds and evaluated its effects on the reduction or inhibition of Aβ, the major cause of neurodegeneration in AD. Theasaponin E1 was found to significantly reduce tau phosphorylation by suppressing and reducing the expression of genes and the activities of various kinases involved in hyperphosphorylation of tau proteins, which leads to the formation and aggregation of NFTs associated with Aβ production and AD pathogenesis. We used SHY-5Y neuroblastoma cells to investigate the inhibitory effects of theasaponin E1 on tau phosphorylation by inhibiting or suppressing the expression levels or activities of various kinases involved in this process. Theasaponin E1 dose-dependently reduced the expression of genes and the in vitro activities of various kinases to varying extents (i.e., GS3β, CDK5, JNK, CAMII, and ERK). In addition, the mRNA expression and in vitro enzymatic activities of the phosphatases PP1 and PP2A were increased dose-dependently. Expression level of various genes that are directly involved in AD causation and pathology were investigated under the influence of theasaponin E1. The results showed that theasaponin E1 dose-dependently suppressed the expression level of genes in SHY-5Y neuroblastoma. The RT-qPCR results showed that PES1, PES2, IDE, EPOE4, and PCALM were significantly downregulated. Furthermore, the expression levels of IDE and TREM 2 were dose-dependently enhanced by theasaponin E1. We evaluated the therapeutic potential of theasaponin E1 upon the suppression and inhibition of inflammatory cytokines, such as IL-1beta, IL10, and TNF alpha, and inflammation-promoting NF-kB pathway in glial cells. After treatment with theasaponin E1, the inflammation level was decreased in glial neuro cells due to the inhibitory effects of theasaponin E1 on the secretion and quantities of the proinflammatory cytokines, IL-1beta, IL10, and TNF alpha. We also evaluated the effects of theasaponin E1 on the inflammation-promoting Nf-kB pathway. Theasaponin E1 dose-dependently reduced the expression levels of IL-1B and NF-kB.

Natural products that can attenuate Aβ, hyperphosphorylation of tau and neuroinflammation are vital products for the prevention or treatment of AD. The green tea bioactive natural products have medicinal and pharmacological effects, including anticancer, antidiabetic, anti-obesity, antiangiogenic, and antimicrobial activities [66,67,68,69]. It has also been found to be effective in functioning of the human brain, healing of liver injury, and enhancing immunity [70,71]. In our previous study, we reported that theasaponin E1 reduced Aβ by inhibition of the amyloidogenic processing of APP and activation of ADAM10 and NCT [72] In the present study, we demonstrated that the green tea seed isolate, theasaponin E1, has a great potential for ameliorating neurotoxic Aβ by reducing p-tau and neuroinflammation.

## 4. Materials and Methods

### 4.1. Process of Theasaponin E1 from Isolation and Purification

Defatted green tea seed (with n-hexane) powder was extracted by continuous refluxing at 60 °C for 6 h in 70% ethanol. The green tea ethanolic crude extract was obtained after filtration and concentration of the above material using jet filter and rotary evaporator. The extract was processed for saponin extraction by first extraction with a butanol and water mixture and then extracting by using nonpolar macroporous resin (D101). A 20 g extract dissolved in 100 mL distilled water was eluted by the resin column first with 0.4 N NaOH and, after neutralization of the column and solution with HCl eluted with 100% ethanol, resulted in a saponin-rich extract. This saponin-rich extract was then purified by preparative high-performance liquid chromatography (HPLC). Elution with a C18 column was done first with 10% MeOH, followed by elution with 60% MeOH, and finally with 100%. MeOH was used to obtain the saponin mixture. Pure saponin (Theasaponin E1) was then isolated from this fraction using a preparative high-performance liquid chromatography (HPLC) system with the following conditions.

HPLC (Shimadzu Co., Kyoto, Japan) equipped with a photodiode array (PDA) detector. The extract was separated on a Luna C-18(2) reverse phase column (250 mm × 21.2 mm, 15 µm; Phenomenex, Inc., Torrance, CA, USA) at 35 °C. Solvent A was methanol and solvent B was distilled water containing 0.1% formic acid. The nonlinear gradient system used was A/B (74:26) to A/B (74.8:25.2) at 33.5 min. to A/B (100:0) for 2 min., followed by holding A/B (100:0) for 10 min. and then A/B (74:26) for 12 min. Components were detected at 210 nm. A flow rate of 7 mL/min. was used.

LC-MS and NMR were used for identification and characterization of the crude and purified isolated saponin.

### 4.2. MTT Assay

SH-SY5Y (ATCC^®^ CRL-2266™) human neuroblastoma cells and U-87 MG glioblastoma (ATCC^®^ HTB-14™) cells were used in the present study. The SH-SY5Y cell type is a good model to investigate neurodegeneration due to pathologies including taupathy and amyloidosis, whereas HTB glioblastoma is a good model for neuroinflammation. Cells were purchased from ATCC and cultured in the media specified by ATCC (EMEM), supplemented with 10% FBS. Safe nontoxic concentration level of saponin to SHY-5Y and U-87 MG cells was determined using an MTT assay. In a 96-well plate, cells were cultured as 1 ×10^4^ cells/well and incubated at 37 °C for 24 h in a humidified 5% CO_2_ incubator. Various concentrations of theasaponin E1 were used for treating the cells in the respective wells of the 96-well plates, and after treatment incubation was continued for 24 h. Wells containing non-treated cells were used as control. After that, 0.5% MTT solution was added to both the treated and non-treated cells and incubated further for 4 h. The MTT- containing media was aspirated from each well and DMSO was added, which resulted in the appearance of a purple color. Absorbance of each well at 540 nm was measured using a microplate reader. Percentage cell viability was calculated and saponin concentrations with more than 90% cell viability were selected for further use in the study. Each experiment was performed in duplicate.

### 4.3. qRT-PCR Analysis

To determine the effects of saponins on the activation or inhibition of AD-related genes and various kinases and phosphatases involved in tau phosphorylation or dephosphorylation (GSK-3β, CDK5, JNK, MAPK, ERK1/MARK, CaMKIIα, PP1, and PP2A), we treated the neuroblastoma cell lines with saponins and evaluated their effects on the expression levels of genes using RT-PCR. Similarly, the effects of saponins on the inhibition of the inflammatory and AD pathogeneses increasing gene Nf-kB and cytokine IL-1B were determined using glial cells, U-87 MG. The expression of the genes was measured and normalized to that of the control (actin). In addition, the expression of each gene following saponin treatment was presented in the form of gel bands. EMEM cells culture media with 10% FBS were used for culturing SH-SY5Y cells in 6-well plates. Cells after attachment were then treated with different concentrations of pure saponins and incubated for 24 h. The control and theasaonin E1-treated cells were then processed for RNA isolation using the specified kit (Sigma Aldrich), followed by DNase treatment. A NanoDrop 2000 spectrophotometer was used for quantification of the extracted total RNA. A total of 500 ng of the isolated RNA was then reverse transcribed to cDNA by the Revert Aid Premium First Strand cDNA Synthesis Kit (Thermo Fisher Scientific). cDNA was then amplified by qRT-PCR using gene-specific primers. The relative expression of each gene was normalized and calculated using *β-actin* as the control gene. The amplified PCR products were then subjected to gel electrophoresis to visualize the DNA bands. The RT-PCR experiments were repeated at least twice.

### 4.4. Quantification of the In Vitro Activities of Kinases and Phosphatases

The effects of saponins on various Tau phosphorylating kinases (GSK-3β, CDK5, JNK, MAPK, ERK1/MARK, CaMKIIα) and dephosphorylating phosphatases (PP1 and PP2A) were measured with ELISA using the specific ELISA Kits (abcam) for each kinase and phosphatase, following the manufacturer’s instructions. SHY-5Y cells were subjected to treatment with saponins after growing in 6-well plates and incubated for 24 h. After incubation, the cell’s lysate was prepared for each concentration and control wells of cells and processed in 96-well microplates for further experiments following the kit’s instruction for each enzymatic assay. Absorbance was recorded at 450 nm with an ELISA microplate reader. Data analysis and comparison were done according to the manufacturer’s instructions.

### 4.5. Effects of Saponins on Proinflammatory Cytokines and Related Pathways in Glial Cells

The effects of saponins on the inhibition or suppression of AD pathogenesis, increasing inflammatory and cytokines, i.e., IL-1B and TNF alpha, were analyzed and quantified in glial cells using specific kits(abcam). U-87 MG cells were cultured in 6-well plates as treated and control groups. Cells in treated groups were treated with various safe concentrations of saponins. Cells were harvested and lysate was prepared and processed in 96-well microplates for further experiments. Absorbance was recorded at 450 nm with an ELISA microplate reader. Data analysis and comparison were done according to the manufacturer’s instructions.

### 4.6. Determination of the Levels of Aβ and Phosphorylated Tau (P-Tau) Using ELISA

The effects of saponins on the expression levels of AD-related genes, *p*-tau and Aβ(1–42), were measured using specific ELISA kits (Amyloid beta 42 Human ELISA Kit Invitrogen and Tau (Total) Human ELISA Kit) following the manufacturer’s instructions. SHY-5Y cells were cultured under the stated conditions in presence and absence (control) of various nontoxic concentrations of saponins. After treatment and incubation with saponins, lysate was prepared and processed in 96-well microplates for the experiments following the kit’s instructions. Absorbance was measured at 450 nm using an ELISA microplate reader. Data were analyzed and compared according to the manufacturer’s instructions.

### 4.7. Quantification of Phosphorylated Tau (P-Tau) in SH-SY5Y Cells Using Western Blotting

To determine whether saponin treatment, after suppression of the AD-promoting genes, affects the phosphorylation of tau, we extracted proteins from SH-SY5Y neuroblastoma cells after treatment with different safe doses of saponins and carried out western blot analysis. Specific p-tau primary antibodies were used for capturing and quantifying p-tau.

### 4.8. Quantification of Proinflammatory Cytokines in Glial Cells

The concentrations of TNF-α, IL-1 beta, and IL-6 in glial cells were measured using specified ELISA kits. Cells were cultured in 6-well plates, as described above, in their respective media and, after attachment, treated with various concentrations of theasaponin E1. Concentrations of inflammatory cytokines in the cell were measured by processing the cell’s lysate of the treated and control wells following the kit’s instructions.

## 5. Conclusions

In the present study, we revealed that theasaponin E1 could be a therapeutic natural product for the treatment and prevention of AD. Theasaponin E1 was found to reduce NFT by decreasing p-tau levels and Aβ formation via the suppression of kinase protein expression and activity, and AD pathology-promoting genes and cytokines. Further studies and clinical trials are, however, required to incorporate theasaponin E1 in health supplements and pharmaceutical formulations.

## Figures and Tables

**Table 1:** Primer sequences used for qRT-PCR analysis of various genes.

Primers	Forward	Reverse
GSK-3β	5′-GGAACTCCAACAAGGGAGCA-3′	5′-TTCGGGGTCGGAAGACCTTA-3′
CDK5	5′-CGCCGCGATGCAGAAATACGAGAA-3′	5′-TGGCCCCAAAGAGGACATC-3′
JNK1	5’-AACTCTTTGACGCTGCTTGC-3’	5’-TGAAGCACTGTGCCTTTACC-3’
MAPK	5′-CCAACTCCTGCCTCCGCTCTA-3′	5′-CCGCCAAAATAACCGATGTGATAC-3′
ERK1/MARK	5’-CGCTTCCGCCATGAGAATGTC-3′	5’-CAGGTCAGTCTCCATCAGGTCCTG-3’
CaMKIIα	5′-AGGAGGAAACTGAAGGGAG-3′	5′-CAGGGTCGCACATCTTCGTG-3′
PP2A	5′-GAGGGTACTACTCTGTGGAGAC-3′	5′-CCGGCTTTCGTGATTTCCT-3′
PP-1	5′-TCCATGGAGCAGATTAGACG-3′	5′-GCTTTGGCAGAATTGCGG-3′
APP	5′-TCAGTTTCCTCGGCAGCG-3′	5′-GCACCAGTTCTGGATGGTCA-3′
PESN1	5′-GCACCGTTGTCCTACTTCCA-3′	5′-CCATGCAGAGAGTCACAGGG-3′
PESN2	5′-GCGGCAGAGCAGGCATTT-3′	5′-AGGTGAAGAGGAACAGCAGC-3′
EPOE4(E4)	5′-GGATGGGGAGATAAGAGAAGAC-3′	5′-CGCAGGTAATCCCAAAAGCG-3′
IDE	5′-CAAGCAGGAAGCGTTTGCG-3′	5′-CAACCTGGTAGTTCCCACACA-3′
TREM2	5′- TTCCCACCCACTTCCATCCTT-3′	5′-AGCAGTGTTCAGGCAGAGTAG-3′
IL-1B	5′- AACAGGCTGCTCTGGGATTC-3′	5′-TTTGGTCCCTCCCAGGAAGA-3′
PICALM	5′-GCAGCTGCCTGTTCCTCTTA-3′	5′-GCAGCTGCCTGTTCCTCTTA-3′
β-actin	5′-CCTCGCCTTTGCCGATCC-3′	5′-GGATCTTCATGAGGTAGTGAGTC-3′

## Data Availability

Data are under use for research purpose; however, they will be provided on proper request to the corresponding author.

## Sample Availability

Samples of the compounds are available from the authors.
